# Multi-Regional Investigation of the Relationship between Functional MRI Blood Oxygenation Level Dependent (BOLD) Activation and GABA Concentration

**DOI:** 10.1371/journal.pone.0117531

**Published:** 2015-02-20

**Authors:** Ashley D. Harris, Nicolaas A. J. Puts, Brian A. Anderson, Steven Yantis, James J. Pekar, Peter B. Barker, Richard A. E. Edden

**Affiliations:** 1 The Russell H. Morgan Department of Radiology and Radiological Science, The Johns Hopkins University, Baltimore, Maryland, United States of America; 2 F. M. Kirby Center for Functional Brain Imaging, Kennedy Krieger Institute, Baltimore, Maryland, United States of America; 3 Department of Psychological and Brain Sciences, The Johns Hopkins University, Baltimore, Maryland, United States of America; Brown University, UNITED STATES

## Abstract

Several recent studies have reported an inter-individual correlation between regional GABA concentration, as measured by MRS, and the amplitude of the functional blood oxygenation level dependent (BOLD) response in the same region. In this study, we set out to investigate whether this coupling generalizes across cortex. In 18 healthy participants, we performed edited MRS measurements of GABA and BOLD-fMRI experiments using regionally related activation paradigms. Regions and tasks were the: occipital cortex with a visual grating stimulus; auditory cortex with a white noise stimulus; sensorimotor cortex with a finger-tapping task; frontal eye field with a saccade task; and dorsolateral prefrontal cortex with a working memory task. In contrast to the prior literature, no correlation between GABA concentration and BOLD activation was detected in any region. The origin of this discrepancy is not clear. Subtle differences in study design or insufficient power may cause differing results; these and other potential reasons for the discrepant results are discussed. This negative result, although it should be interpreted with caution, has a larger sample size than prior positive results, and suggests that the relationship between GABA and the BOLD response may be more complex than previously thought.

## Introduction

BOLD fMRI is widely used to study brain activity either with or without explicit stimuli. The BOLD signal results from a combination of physiological factors, including cerebral hemodynamics, metabolic responses and oxygenation that provide a surrogate measure of the underlying neural activation [1,2]. In traditional task-based BOLD-fMRI studies, a stimulus is presented to increase brain activity. In response to this stimulus, there is an increase in regional oxygen consumption. This is accompanied by an increase in cerebral blood flow (CBF) and cerebral blood volume (CBV) that is greater than that required to fulfill the additional oxygen consumption, resulting in a decrease of local deoxyhemoglobin which impacts MR signals as the BOLD effect. Understanding the physiology of the BOLD signal, specifically the neural activation that it reflects and the relationship between this activation and signaling, and the metabolic and hemodynamic response, will improve its interpretation and application. Methods to investigate CBF and CBV as a function of cerebral activation can interrogate some of the hemodynamic components of the BOLD signal [3,4] and calibration techniques can be applied to investigate cerebral metabolic oxygen consumption [2], however these methods do not provide information on the underlying neurochemical and signaling mechanisms involved in brain activation.

γ-Aminobutyric acid (GABA) is the primary inhibitory neurotransmitter in the human brain and is involved in the regulation of cortical activity [5–7]. Local GABAergic inhibition modulates metabolically demanding glutamatergic excitation [1,5,8]. The balance between excitatory and inhibitory neurotransmission therefore impacts local hemodynamics, and is reflected in the BOLD signal [1,7]. In addition to the presynaptic neurotransmitter pool of GABA, there is a larger metabolic GABA pool [5]. The metabolic GABA pool is located throughout the cell cytoplasm and is therefore hypothesized to be involved in metabolism [9]. Edited MRS can be used to measure GABA *in vivo* but cannot differentiate between pools and is therefore interpreted as an index of overall inhibitory tone rather than current neuronal inhibitory activity [5]. Differences in measured GABA have implicated its role in neurological and psychiatric conditions as well as in behavioral tasks [5,6,10–13].

The relationship between GABA concentration and BOLD activation has been examined previously in healthy subjects by correlating the magnitude of the task-related BOLD response to baseline GABA concentrations [12–17]. The negative BOLD response in the anterior cingulate during an emotional task has been shown to be positively correlated with GABA concentration [17]. The task-positive BOLD signal has been shown to correlate negatively with GABA concentration in the occipital cortex [13–16] and in the primary motor cortex [12]. However, each of these studies measured GABA in a single region, often with relatively small sample sizes (n < 15). Therefore, it is currently unclear if the correlations between GABA concentration and BOLD response can be generalized across the brain.

To test the hypothesis that GABA plays a fundamental role in controlling activation throughout the brain, in this study, GABA-edited MRS data were acquired from 5 voxels (occipital cortex (OCC), auditory cortex (AUD), sensorimotor cortex (SM), frontal eye field (FEF) and dorsolateral prefrontal cortex (DLPFC)) and examined for correlations with BOLD-fMRI data during 5 tasks to elicit activity in these regions. These regions were chosen to reflect a range of cognitive function from low- to higher-level: primary sensory; primary motor; motor control; and higher cognitive processing.

## Materials and Methods

### Recruitment, Ethics and General Study design

All subjects provided informed, written consent prior to the studies. This study was approved by The Johns Hopkins School of Medicine Institutional Review Board.

All scanning was performed on a 3T Philips Achieva scanner (Philips Healthcare, The Netherlands) using a 32-channel head coil. Eighteen healthy volunteers (11F/7M, 27.8 ± 4.0 yrs) were recruited to participate in two scanning sessions: for both sessions, a T1-weighted anatomical image (MPRage, TR/TE = 8 ms/3.7 ms, 1 mm^3^ isotropic voxels) was acquired for voxel placement and segmentation. This was followed by GABA-edited MRS acquisitions and then BOLD fMRI. One session acquired GABA-edited MRS data and corresponding functional tasks for the occipital cortex (visual grating viewing task), auditory cortex (auditory white noise task) and sensorimotor cortex (finger tapping task) and the other session included acquiring GABA data from the FEF (FEF-activating saccade task) and the DLPFC (working memory task). The order of the two sessions was randomized across subjects.

### MRS acquisition and analysis

**Fig 1:**
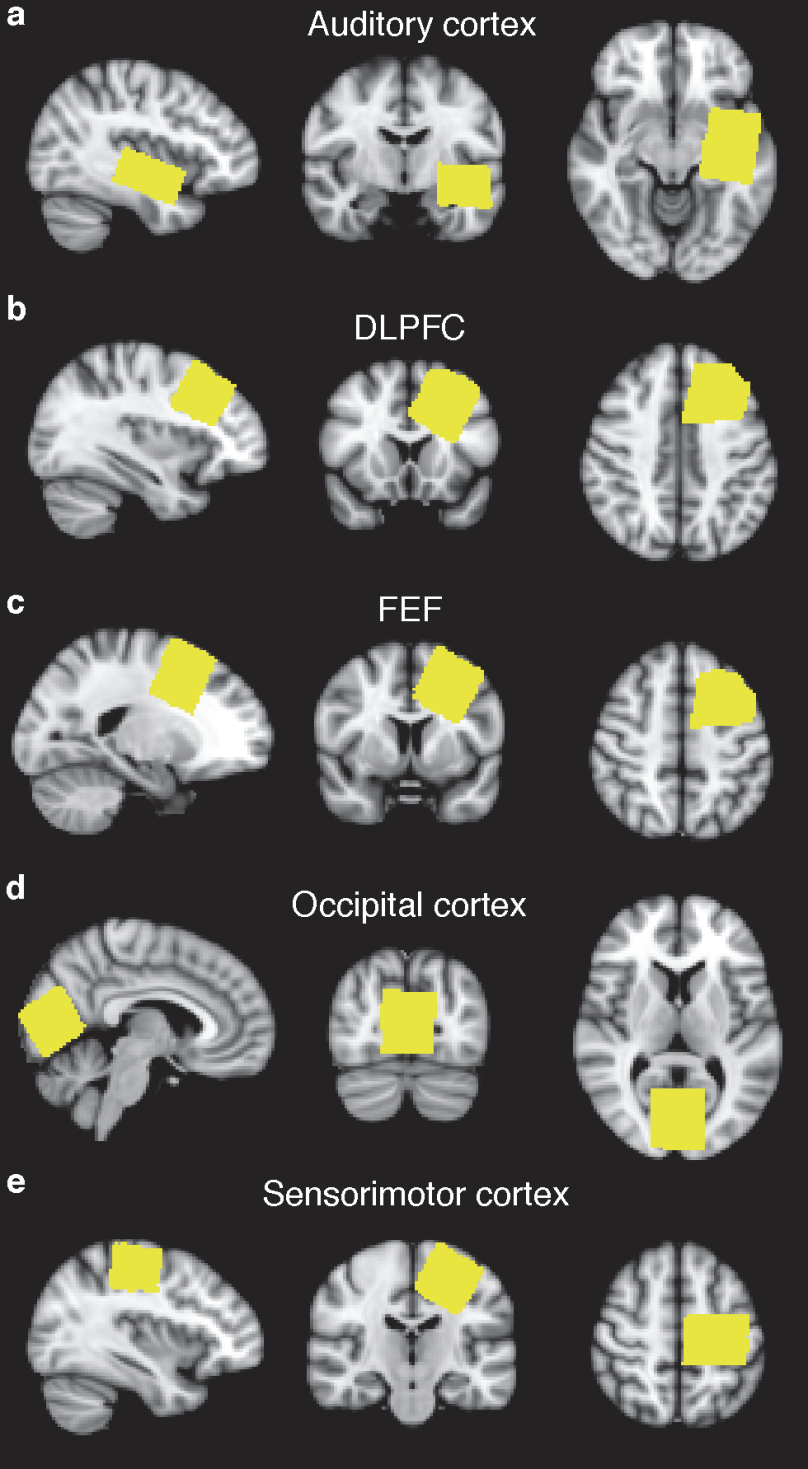
MRS voxel placement. MRS voxel mask images for one subject, transformed into standard space and overlaid on the MNI standard-space atlas, for all five locations: (a) auditory cortex; (b) dorsolateral prefrontal cortex; (c) frontal eye field; (d) occipital cortex; and (e) sensorimotor cortex.

GABA-edited MRS data were acquired using the MEGA-PRESS experiment [18], with 14 ms sinc-Gaussian editing pulses applied at 1.9 ppm in the ‘ON’ condition and 7.46 ppm in the ‘OFF’ condition. Acquisition parameters included: TR/TE = 2s/68 ms; 320 transients with ON-OFF scans alternating every 2 transients; a 16-step phase cycle (with steps repeated for ON and OFF); 2048 data points acquired at a spectral width of 2 kHz; VAPOR water suppression [19]. Unsuppressed water data was also acquired for quantification using the same acquisition parameters including the TE = 68 ms, 16 transients and water suppression turned off. With the exception of the auditory voxel, which was 4 × 3 × 2 cm^3^, all voxels were 3 × 3 × 3 cm^3^. Voxel locations from one subject are shown in Fig. 1.

Quantitative analysis was performed using the ‘Gannet’ program (version 2.0; [20]). All time domain data were frequency- and phase-corrected using spectral registration [21], filtered with a 3 Hz exponential line broadening and zero-filled by a factor of 16. The 3 ppm GABA peak in the difference spectrum was fit using a five-parameter Gaussian model and quantified relative to water (fit with a Gaussian- Lorentzian model) in institutional units (i.u.); secondary quantification was performed relative to the creatine (Cr) peak (from the Cr integral of a two-Lorentzian model of Cr and choline in the OFF spectrum).

A binary mask of the MRS voxel was created, with the same imaging matrix as the T1-weighted anatomical image, using the SVMask tool. Voxel tissue content was determined using FAST [22] to segment the image and apply this segmentation to the overlaying the voxel mask. The tissue fraction (voxel fraction of white matter and grey matter, f_WM_ and f_GM_, respectively) was then used to normalize the GABA concentration (relative to water) according to: GABAcorr = GABA/(f_WM_ + f_GM_).

### BOLD acquisition and analysis

Across the two MR sessions, five fMRI acquisitions were performed, each with a different task: visual grating; auditory white noise; sensorimotor finger tapping; saccades; and working memory. Stimuli were presented using PsychToolBox3 [23,24] in Matlab (2012, Mathworks, Natick MA, USA). The visual task was based on that described in reference [15]—a maximum contrast vertical grating in the lower left visual field, subtending ~4.5° horizontally and vertically, presented on a mean luminance background. The upper right corner of the stimulus was 0.5° horizontally and vertically from a center fixation cross hair and the grating consisted of 12 cycles. The isoluminant background and cross hair was presented between trials. The auditory stimulus was white noise presented using MR compatible headphones (Model S14, Sensimetrics, Malden, MA, USA), and a crosshair for fixation was displayed on the screen throughout. For the auditory and visual tasks, participants were requested to respond on a button box (in left hand) at the end of the stimulus to monitor attention. The sensorimotor task consisted of visually cued sequential finger-to-thumb tapping of the right hand (1 cycle per trial) with a cross hair for fixation between trails. Compliance was visually monitored. For the saccade task, participants had to follow a cross hair by making eye movements. Between trials, the crosshair was at the center of the screen. During a trial it appeared horizontally to one side, back in the center and then on the other side; the ordering of the starting side was counterbalanced. Compliance was monitored using an eye tracker (MRA Inc., Washington PA, USA). All four of these tasks consisted of 2-second trials and inter-stimulus intervals of 6, 8 or 10s (uniform distribution, psuedo-randomly sampled) for 36 trials. The fifth task was a working memory task, consisting of 30s blocks of alternating 2-back and 0-back conditions. Training for all tasks was performed prior to scanning.

BOLD data were acquired using a gradient-echo EPI sequence, with parameters: TR/TE = 2.5s/30 ms; 144 repetitions; 3 × 3 × 3 mm^3^ isotropic voxels; 4 dummy scans. Data were analyzed using FEATv6, in FSL according to the following analysis pipeline: motion correction (McFlirt [25]); slice-timing correction; skull-stripping (BET [26]); spatial smoothing using a 6 mm Gaussian kernel; mean-based intensity normalization of all volumes; and temporal filtering (Gaussian-weighted least-squares straight line fitting, σ = 30.0s). A general linear model (GLM) analysis was then applied, accounting for local autocorrelations using FILM [27] and statistical images were thresholded using clusters determined by a Z > 2.3 and a cluster-corrected significance threshold of p = 0.05 [28]. Registration of functional data to the T1-weighted anatomical image and subsequently to standard space was performed using FLIRT [25,29]. In keeping with previous studies [13,15,16], the primary measure of BOLD activation was then quantified by the signal change of the peak fMRI voxel, within the corresponding MRS voxel. Secondary metrics quantifying the BOLD response included: mean BOLD activation across the MRS voxel; mean BOLD activation across the subject-specific significant activation within the anatomical region of interest; the peak BOLD activation within the anatomical region of interest; and the mean BOLD activation across the anatomical region of interest. Anatomical regions of interest were defined using the atlases available in FSL and were: primary visual cortex; primary auditory cortex; primary sensorimotor cortex; middle frontal gyrus; and a union mask of the middle frontal gyrus and inferior frontal gyrus pars opercularis masks, for the visual, auditory, finger-tapping, saccades and working memory tasks, respectively. These regions were then registered to each individual native space.

### Statistical Methods

The relationship between BOLD activation and GABA was examined using the Pearson correlation coefficient of individual’s paired GABA and BOLD measurements. The Bonferroni-corrected threshold for statistical significance was set at p = 0.01 to account for the 5 regions investigated. Relationships to secondary measures (GABA quantified relative to Cr, and secondary BOLD measures, see section 2.3) and within sex-separated subgroups were considered using the same statistical methods. Post-hoc Bayes factor (BF) analysis [30] was performed to evaluate the strength of evidence in support of the null hypothesis and/or the alternative hypothesis.

In order to consider the results of the current study in combination with previous studies examining the correlation between GABA and BOLD activation in the occipital cortex, a meta-analysis using the Hunter-Schmidt method [31] was performed. Data from this study and those of Muthukumaraswarmy [15,16], Donahue [14] and Violante [13] were included.

## Results

**Fig 2:**
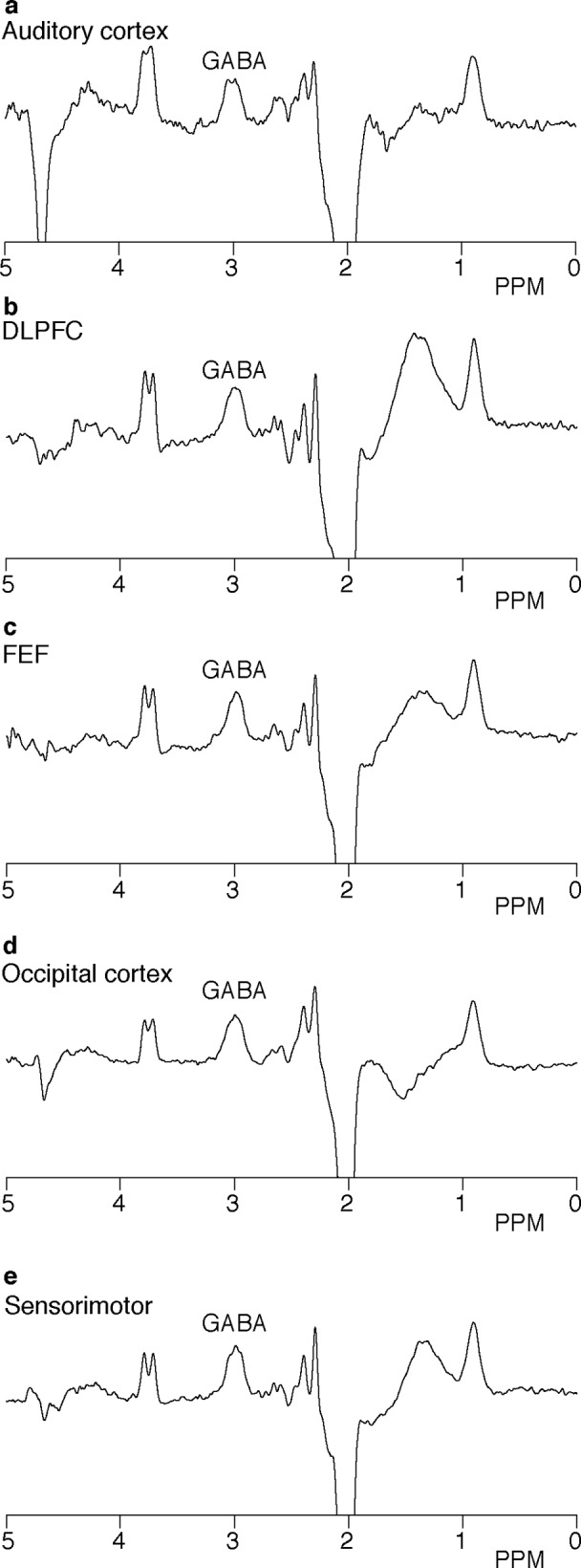
GABA-edited MRS. Representative spectra for all regions from one subject: (a) auditory cortex; (b) dorsolateral prefrontal cortex (DLPFC); (c) frontal eye field (FEF); (d) occipital cortex; and (e) sensorimotor cortex. These spectra are from the same subject as the voxel masks shown in Fig. 1.

**Fig 3:**
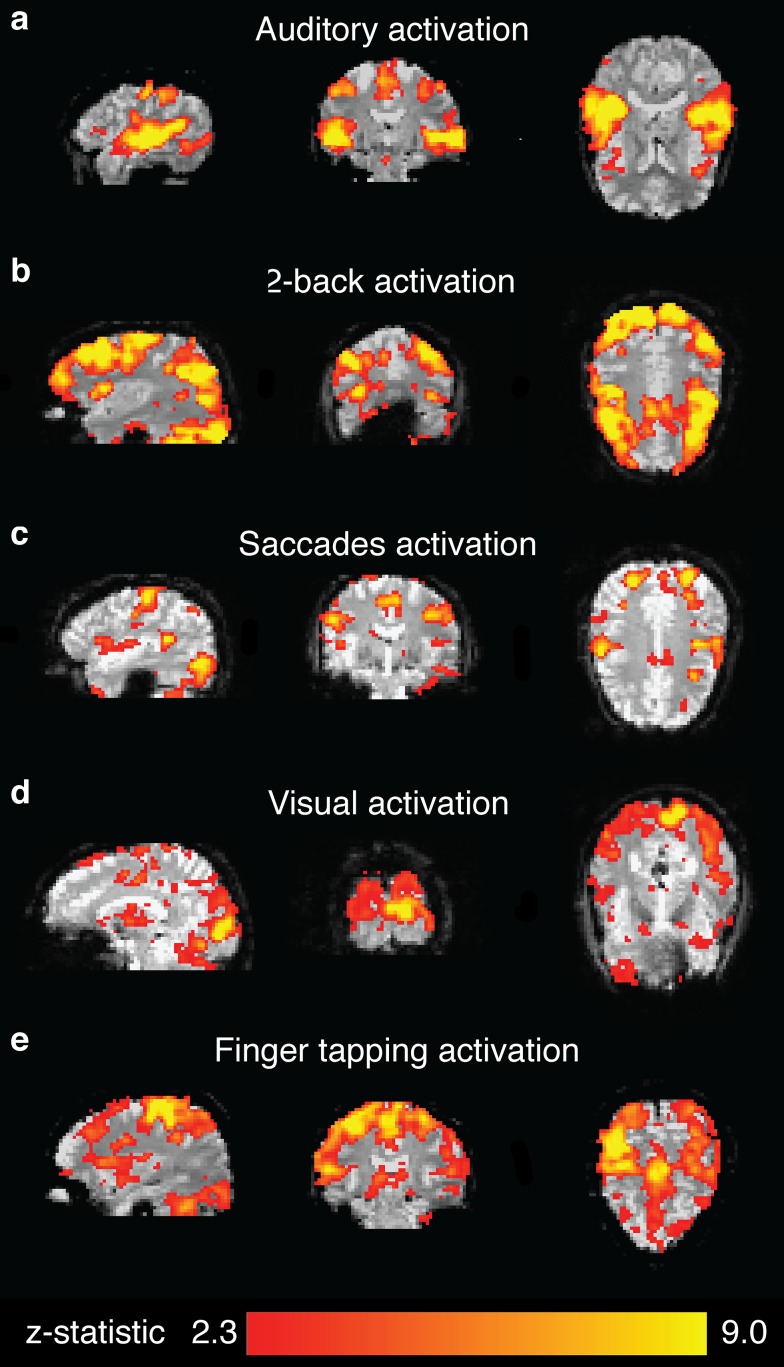
Functional MRI. Single subject Z-statistic maps showing BOLD activation for each task for the same subject as Figs. 1 and 2. Amongst other regions, the auditory task activated perisylvian primary auditory cortex (a), the working memory task activated DLPFC (b), the eye-tracking task activated FEF (c), the visual task activated occipital visual cortex (d); and the finger-tapping task activated primary motor and somatosensory cortices (e).

GABA spectra that were not of acceptable quality (*i*.*e*., as evidenced by a drift or jump in frequency by 20 Hz or more during the MRS acquisition, or subtraction artifact that could not be resolved with frequency correction) were removed from analysis along with the corresponding BOLD measurements. Two auditory datasets, two sensorimotor datasets, one occipital dataset, one FEF dataset, and two DLPFC datasets were excluded for these reasons. Example GABA spectra from each location from a single subject are shown in Fig. 2. One sensorimotor dataset for the finger tapping task and one dataset for the visual task did not produce subject-level significant activation and were therefore excluded from the secondary analyses that quantified the BOLD using the region of significant activation. The BOLD z-statistic maps of task-related activation for one subject is shown in Fig. 3.

**Fig 4:**
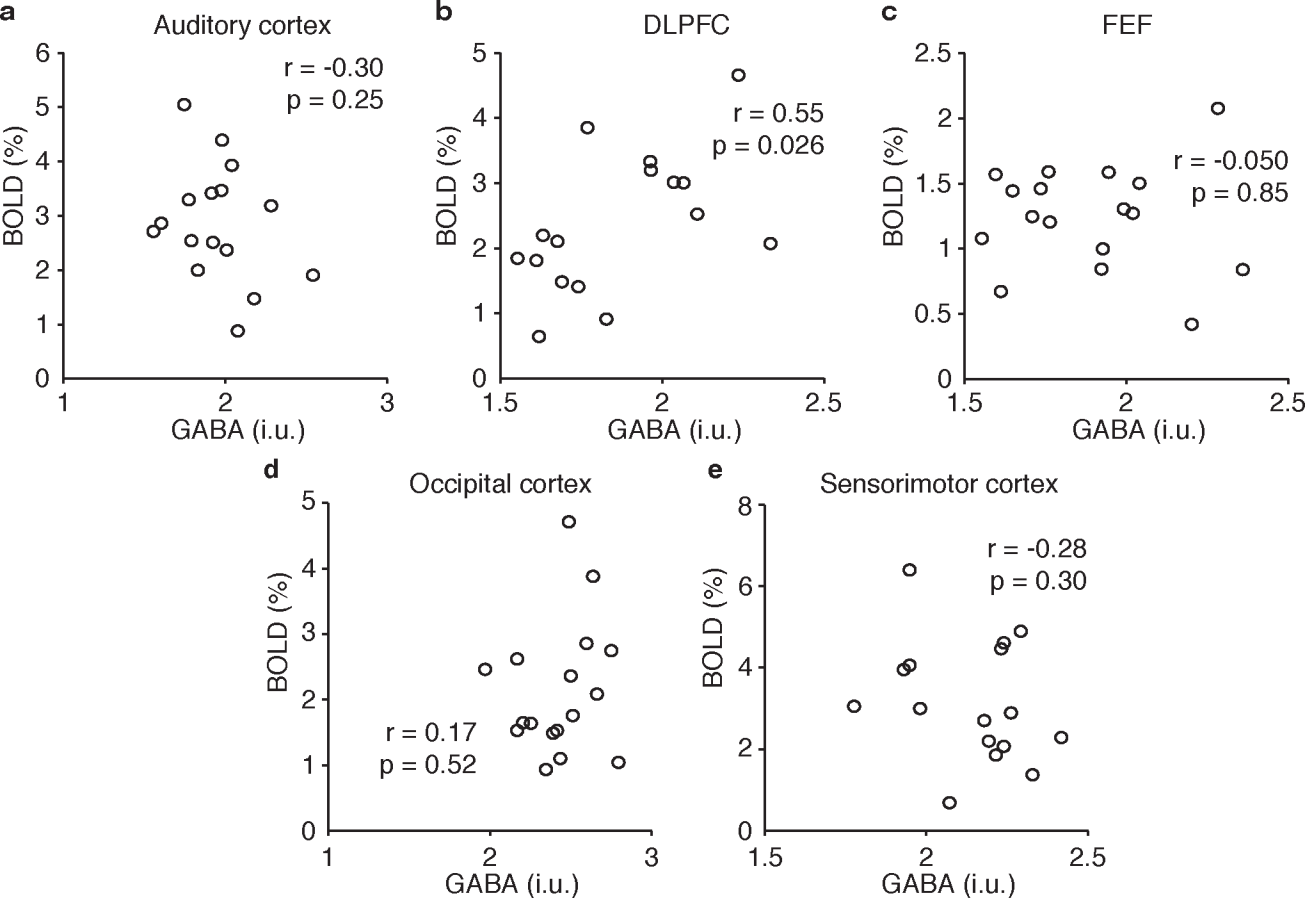
Inter-individual GABA-BOLD correlations. GABA, quantified in institutional units, and BOLD signal change, as defined by the peak voxel in the MRS voxel, are plotted for all five regions. To correct for multiple comparison, a threshold of significance of p = 0.01 was set; p > 0.02 for all five comparisons. The DLPFC result, which approaches significance, has a positive slope in contrast to previous results.

Table 1 summarizes the group average measured GABA and peak BOLD response within the MRS voxel for each region. The inter-individual relationships between the peak BOLD signal within the MRS voxel and GABA concentration are shown in Fig. 4. The only trend is a positive correlation in the DLPFC, but this does not maintain significance after accounting for multiple comparisons. Similarly, none of the secondary analyses show a significant correlation (*i*.*e*., either when quantifying GABA with respect to Cr, or when quantifying the BOLD response by the mean across the MRS voxel, the mean across the region of significant activation, the peak voxel within the anatomical mask or the mean activation across the anatomical mask) after accounting for multiple comparisons. Table 2 summarizes the calculated correlation coefficient and p-values for all regions and comparisons, including the primary analysis and subsequent secondary analyses using GABA:Cr measures and secondary BOLD analysis (peak response with in anatomical region, average activation within an anatomical region, average activation across the MRS voxel, average activation across region of significant activation). Secondary analysis by sex did not reveal any correlations and pooling all regional data did not produce any trends (r = 0.078, p = 0.49). Bayes factors for the AUD, DLPFC, FEF, OCC and SM voxels were 0.36, 2.1, 0.19, 0.23 and 0.33, respectively. For the AUD, FEF, SM and OCC voxels the null hypothesis, (i.e. that there is no correlation between measured GABA and BOLD activation) is supported by the Bayes factor. The Bayes factor for the correlation between GABA and BOLD in the DLPFC was 2.1, that shows anecdotal evidence in support of this correlation. The meta-analysis combining the results of the present study with four previous studies [13–16] gives a 95% credibility interval of 0.086 to -0.97. Since this range includes zero, the presence of a correlation between GABA and BOLD in the occipital cortex is not supported at a credibility level of 95%.

**Table 1:** Group average (standard deviation) GABA and peak BOLD response within the MRS voxel for all regions and divided by gender.

	region	GABA:water (i.u.)	GABA CV (%)	Peak BOLD(%)	BOLD CV(%)
All subjects					
	Auditory	1.95 (0.25)	12.77	2.88 (1.07)	37.34
	DLPFC	1.86 (0.24)	12.96	2.38 (1.08)	45.39
	FEF	1.89 (0.24)	12.92	1.24 (0.40)	32.61
	Occipital	2.43 (0.23)	9.28	2.14 (1.01)	47.39
	Sensorimotor	2.14 (0.18)	8.25	3.16 (1.48)	46.93
Female					
	Auditory	1.96 (0.29)	15.02	2.66 (0.55)	20.50
	DLPFC	1.86 (0.23)	12.15	2.51 (1.24)	49.46
	FEF	1.95 (0.27)	13.79	1.23 (0.48)	38.50
	Occipital	2.44 (0.20)	8.30	1.98 (0.94)	47.40
	Sensorimotor	2.14 (0.20)	9.22	3.18 (1.24)	38.98
Male					
	Auditory	1.94 (0.20)	10.27	3.15 (1.53)	48.41
	DLPFC	1.88 (0.30)	16.05	2.08 (0.59)	28.39
	FEF	1.78 (0.16)	8.85	1.25 (0.27)	21.42
	Occipital	2.41 (0.27)	11.21	2.36 (1.15)	48.56
	Sensorimotor	2.15 (0.16)	7.53	3.12 (1.85)	59.30

**Table 2:** Correlation coefficient (r) and statistical significance (p) for the relationship between resting GABA and BOLD activation.

	BOLD Peak within MRS voxel		BOLD Mean across MRS voxel		BOLD mean across significant activation		BOLD peak within anatomic ROI		BOLD mean across anatomical ROI	
	r	p	r	p	r	p	r	p	r	p
GABA: Water										
Auditory	*-0.30*	*0.25*	-0.34	0.19	-0.22	0.41	-0.22	0.42	-0.13	0.64
DLPFC	*0.55*	*0.026*	0.51	0.041	0.48	0.060	0.49	0.053	0.53	0.035
FEF	*-0.050*	*0.85*	0.12	0.65	0.12	0.64	-0.19	0.46	0.028	0.91
Occipital	*0.17*	*0.52*	-0.097	0.71	-0.30	0.26	-0.15	0.57	-0.16	0.54
Sensorimotor	*-0.28*	*0.30*	-0.36	0.17	-0.10	0.71	-0.44	0.090	-0.28	0.30
GABA: Cr										
Auditory	-0.25	0.35	-0.033	0.9	-0.15	0.57	-0.11	0.69	-0.13	0.63
DLPFC	0.43	0.096	0.4	0.12	0.33	0.21	0.4	0.13	0.41	0.12
FEF	0.14	0.59	0.45	0.071	0.29	0.25	0.14	0.57	0.16	0.54
Occipital	0.19	0.45	-0.16	0.54	-0.29	0.28	-0.14	0.59	-0.27	0.29
Sensorimotor	-0.38	0.14	-0.54	0.031	-0.19	0.50	-0.55	0.028	-0.37	0.16

The primary analysis, correlating the peak BOLD response within the MRS voxel and GABA quantified with respect to water, is shown in italics. The Bonferonni-corrected statistical threshold to account for multiple comparisons (5 regions) is p = 0.01.

## Discussion

No significant correlation was observed between GABA concentration and BOLD activation in any of the five regions. This outcome is at odds with the stated hypothesis and with previously published studies. A trend towards a positive relationship was observed between GABA concentration in the DLPFC and BOLD activation during the working memory task, but this was not significant after accounting for multiple comparisons (*i*.*e*. the five regions studied). None of the secondary analyses showed a trend between GABA concentration and BOLD activation (Table 2). Similarly, the Bayes factor analyses support the null hypothesis (i.e., there is no correlation between GABA and BOLD) in addition to the alternative hypothesis evaluation performed using the correlation coefficient. A Bayes factor of 1/3 to 1 is anecdotal evidence in support of the null hypothesis, and a Bayes factor of 1/3 to 1/10 is substantial evidence in support of the null hypothesis [30], thus the analysis of the correlation coefficients for the AUD, FEF, SM and OCC analysis is consistent with the primary result. For the DLPFC, prior to correction for multiple comparisons the correlation coefficient showed a significant correlation between GABA and BOLD. The Bayes factor (2.13) showed anecdotal evidence in support of the GABA-BOLD correlation in the DLPFC also consistent with the trend towards a correlation as seen prior to correction for multiple comparisons.

### Comparison to prior studies

A meta-analysis was performed to combine results from the current study and the four previous studies that examined the relationship between GABA and BOLD in the occipital cortex. This analysis combines correlation coefficients for different studies in a way that is weighted by sample size. In combination, these studies do not support a relationship between GABA and BOLD at a 95% credibility threshold.

While this study did not replicate the previously observed inverse relationships between GABA concentration and BOLD activation [12–17] it is not, in the strictest sense, a replication of these prior studies, differing in the acquisition and analysis methodologies of both MRS and fMRI. The failure to ‘replicate’ may indicate that the relationship between GABA concentration and BOLD activation is not as strong as originally observed, or that it is being confounded by unidentified system complexity, subtle differences in study design, data acquisition, subjects or insufficient statistical power (*i*.*e*. a false negative). Alternatively, it is possible that prior results were driven by an unrecognized artifact, such that they represent false positives. Below we review prior studies (summarized in Table 3), highlighting experimental differences and similarities.

**Table 3:** Summary of previously published studies showing a correlation between baseline GABA and task-related BOLD activation.

	Northoff et al. 2007 [17]	Muthukumaraswamy et al. 2009 [15]	Muthukumaraswamy et al. 2012 [16]	Donahue et al. 2010 [14]	Stagg et al. 2011 [12]	Violante et al. 2013 [13]
Scanner	Philips	GE	GE	Siemens	Siemens	Siemens
N	12 (initial group = 25, 15 F, 10 M)	12 (all M)	15 (all M)	12 (6 M, 6 F)	12 (6 M, 6 F)	26 (10 M, 16 F)
Age (mean ± std)	33.8, range: 28–42	34.8 ± 4.8	24.2 ± 2.4	30 ± 4	23, range: 21–31	13.1 ± 2.9
R	-0.713 (all pictures)	-0.64	-0.64 (ROI), -0.78 (peak voxel)	-0.70	-0.688	-0.458
P	<0.01	<0.05	< 0.02 (ROI) <0.01 (peak)	0.01	0.01, uncorrected	0.019
BOLD CV (%)	36.3%	33.5%	33.4%	28%	31.3%	28.6%
GABA CV	37.1%	7.6%	15.1%	9.95%	31.2%	9.7%
fMRI						
Paradigm	Picture judge-ment, multiple conditions and timings	1.5–2s/10s, vertical grating, 42 events	2s/18s, vertical grating, 45 events	20s/40s block design flashing checker-board 3 blocks	Motor reaction task, multiple conditions in GLM block design	1.5s-2s/ 10s, circular moving gradient, 30 events
BOLD	Mean across negative BOLD response	Peak response in V1	Peak and mean sig activation in V1	Average across significant activation	Average across group significant activation	Peak voxel in V1
spatial filtering	8 mm	5 mm	5 mm	Unclear/ 0 mm	5 mm	5 mm
MRS						
Region	ACC	Occipital cortex	Occipital cortex	Occipital cortex	Motor cortex	Occipital cortex
Voxel size	17.5 cm3	27 cm3	27 cm3	27 cm3	8 cm3	27 cm3
Method	J-Press	MEGA-PRESS	MEGA-PRESS	MEGA-PRESS	MEGA-PRESS	MEGA-PRESS
Acquisition parameters	TE = 31–229, TR = 2.5s,	TR/TE = 1.8s/68 ms, 3Hz line broadening	TR/TE = 1.8s/68 ms, 3Hz line broadening	TR/TE = 2s/69 ms, 5 Hz line broadening	TR/TE = 3s/68 ms, 2Hz line broadening	TR/TE = 1.5s/68 ms, 4Hz line broadening
Number of averages		512 x 2 acquisitions	512 x 2 acquisitions	192	256	196
MRS quantification	GABA:water	GABA: water (i.u.)	GABA: water (i.u.)	GABA:NAA, NAA:Cr	GABA:NAA, GABA corrected for GM, NAA corrected for tissue	GABA:Cr

Abbreviations: M male; F female.

### Participants

Small sample sizes can lead to insufficient statistical power and results being driving by a few data points or effect inflation [32]. For the most part, previous studies consisted of data from 12–15 young, healthy participants [12,14–17], with relatively narrow age ranges. One other study included 26 participants [13], with a younger age range (7.4–19.7 year). The sample of 18 subjects in the present study my not be enough to overcome power limitations, but it is not inconsistent with the sample size and age range of previous studies. Thus, the null result in this study may indicate effect inflation in previous work.

Both males and females were included in the current study primary analysis. Follow-up analyses by sex did not reveal any differences. Differences in GABA between males and females [10,33] as well as throughout the menstrual cycle have been reported [34,35]. Some of the previous studies examining the relationship between GABA concentration and BOLD activation chose to restrict their study population to only males [15,16,36], while others did not [12–14]. As the GABA-BOLD correlation had been demonstrated in studies including both males and females, we chose to include both in the present study. The paired regional GABA measurements with fMRI task were performed in the same session therefore any within-subject changes in GABA during the menstrual cycle were in principle controlled for; however, the potential for effects that uncouple during the menstrual cycle remain. Nevertheless, when analyzing the data by sex, no differences between males and females were observed.

### fMRI paradigms

The GABA-BOLD relationship has been most extensively studied in the occipital cortex [13–16], however, failure to replicate this relationship has also been reported [37]. The visual task of a vertical grating appearing in the lower left visual field was comparable to that of previous studies [15,16]; however the stimuli timings were slightly different. Stimuli were presented for 2s and the interstimulus interval was jittered in the present study as opposed to a fixed interstimulus interval and stimuli presented for 1.5–2s (then modeled as 2s in the GLM analysis). In the present study, the fMRI paradigm included 36 events while there were 42 in the original correlation study of GABA and BOLD in the occipital cortex [15] and 45 events in the follow-up study by the same group [16]. The increase in SNR with the increased number of events may contribute to the sensitivity of the BOLD measure. However, this increase in SNR by increasing the number of events may not be necessary [13]. By contrast, a block design with a blue and yellow flashing checkerboard stimulus, which would increase the extent of activation has been used [14]. This difference in stimulus and presentation appears to indicate that replicating the exact task is not a requirement in detecting a relationship between GABA concentration and BOLD activation. The spatial blurring in previous studies was not drastically different from that used in the current study (previous studies used a 5 or 8 mm FWHM kernel, compared to the 6 mm kernel applied here), therefore changes in SNR due to spatial blurring are unlikely to explain differing results.

It is more difficult to compare the sensorimotor paradigm here with the previous work [12]. In the present study a simple, sequential finger-tapping paradigm was presented and there was only one regressor of interest in the present analysis. By contrast, the previous study used a motor learning and reaction task that was part of a more complicated multivariate analysis [12].

### BOLD quantification method

Previous studies correlated GABA concentration with the peak BOLD response [13,15,16], the BOLD average across the significant activation within a specified region of interest [16,17] and the mean response across significantly activated tissue [12,14]. None of these analyses explicitly restricted the GABA measurement and the BOLD response to originate from the same anatomical tissue as was performed in the primary analysis here. It is possible, if not likely in some cases, that the region or voxel for the BOLD measurement and GABA voxel do not always completely overlap in previous studies. This is because the BOLD response is generally localized on the surface of the cortex and the MRS voxel is placement includes more than the surface of the brain. For example, it is very difficult to place a 3 × 3 × 3 cm^3^ voxel so as to include all the cortex of V1 without any non-brain tissue. Nevertheless, secondary analyses that did not restrict the BOLD and GABA measurements to originate from the same tissue also did not replicate the central result.

### MRS Acquisition

When comparing data with the literature, differences in data quality must be considered. A visual inspection of all GABA spectra was performed and poor quality spectra were removed. The voxel sizes of 24–27 cm^3^ used in the current study are consistent with the majority of previous studies. A single operator performed all scanning for consistency in voxel placement across participants. Thus differences in the MRS voxel placement itself likely do not explain discrepancies between the results here and the literature.

The coefficients of variation (CV) of the GABA measures shown in Table 1 are less than 15% for all regions for the full cohort, of the same order as four previous studies and substantially lower than 2 previous studies (refer to Table 3). In the present study, the GABA measurement consisted of a single acquisition composed of 320 transients, while previous studies [15,16] averaged two GABA measurements, each of which contained 512 transients. Others using MEGA-PRESS have used a single measurement composed of less than 200 averages [13,14]. One large-CV study (Stagg et al.) traded measurement SNR for greater spatial specificity, and the other (Northoff et al. [17]) used a different (J-PRESS) acquisition strategy. In the *J*-PRESS study [17], over 40% of participants were excluded on the basis of spectral quality. *J*-PRESS has different signal characteristics compared to MEGA-PRESS, as used in the rest of the studies. In terms of anticipated SNR, based upon acquisition time and voxel volume, our study lies somewhere in the middle of the distribution of previous studies, and it is difficult to attribute the current null result purely to GABA SNR.

Reproducibility measurements have shown MEGA-PRESS to be reliable in measuring GABA [38–40]; however, the interaction of individual variability and experimental variability is unclear.

Reduced measurement error may be expected from improvements in the GABA measurement over time. The main area of development has been in post-processing corrections for frequency and phase errors associated with experimental instability. We have shown previously that these errors tend to decrease measured GABA [41]. It is possible that variance associated with experimental instability could lead to spurious correlations. BOLD measurements may also be impacted by instability, tending to reduce the observed BOLD signal change; however, this would tend to produce a positive correlation between GABA and BOLD rather than the observed negative correlation.

### GABA quantification

Generally, previous studies have quantified GABA using the ratio of GABA to another metabolite, either creatine or NAA [12–14,17]. In one instance [12] the GABA and NAA were then differentially corrected for white matter and grey matter tissue fractions. The two studies [15,16] that quantified GABA:water did not correct for tissue fraction though the average tissue and CSF proportions were reported. Here, as a primary measure we reported the GABA:water ratio (to quantify GABA using i.u.) and the tissue fraction was used to correct the concentration. In secondary analyses, GABA was also quantified with respect to creatine without differential findings. It is therefore unlikely the difference in result between the present study and previous studies is a function of metabolite quantification method.

While none of the tasks or acquisitions were rigid replications of previous studies, the secondary analyses largely replicate all previous correlation metrics for GABA and BOLD measurement. There are differences between the present study and previous studies in study design and acquisition but, for example, there appears to be greater differences between the four published studies examining the relationship between GABA concentration and BOLD activation in the occipital cortex (refer to Table 3). For this reason, it seems unlikely that the null result in the present study is a function only of the differences in study design and data analyses compared to previous studies.

### Sources of variability affecting individual differences

The ability to detect inter-individual differences for correlated measures relies on sufficient cohort heterogeneity and measurement accuracy. The CVs in this study were on the same order for the GABA measurements compared to comparable studies (i.e., same acquisition method and voxel size). The BOLD CVs were higher in the present study compared to previous studies. It is not easy to isolate the source of this increase in variance as comparable acquisition, filtering and analysis methods were applied, and recordings to monitor compliance showed that participants were performing tasks. It is possible that other sources of variance impact these measurements obscuring the correlation of interest. Differences in cognitive factors, physiology, morphology, situational factors, and genetic differences may all generate inter-individual differences [42,43].

The BOLD response itself can display substantial variability with one study showing 25% variability of activation amplitude with somatosensory stimuli [44] and others finding a 24% variability in an inhibition task and 14% in a visual task [45]. Inter-individual differences in BOLD can be driven by vascular differences [46] and investigations into the relationship between GABA concentration and the CBF response to stimuli have shown conflicting results [14,36]. In one study, CBF was measured using a pulsed ASL acquisition with a single inversion and a positive relationship between GABA and CBF was shown [14]. However, a follow-up study using pseudo-continuous ASL and multiple inversion times found no relationship between GABA and CBF when using a single inversion pulse and a negative relationship was detected between GABA and CBF as measured using multiple inversion times [36]. While technical differences exist, this inconsistency in the relationship between GABA and the CBF response may explain discrepancies in observing correlations between the BOLD response and GABA concentrations, particularly since CBF is a dominant factor in the BOLD response.

A standard MEGA-PRESS GABA-edited acquisition and quantification method was used to measure GABA in this study [11,18,20]. This is, however, subject to macromolecular contamination of the GABA peak due to co-editing of homocarnosine and the macromolecule resonance at 1.7 ppm [6]. For this reason, GABA measurements are often referred to as GABA+. Variability in the macromolecular contribution of measured GABA due either to individual differences or experimental error (*i*.*e*., resulting from drift) may have a more substantial effect than generally expected [47,48] such that the macromolecular component affects individual differences. While there are methods to suppress macromolecules [49–51], these methods are not yet standard and the efficacy of macromolecular suppression and the susceptibility to experimental error resulting from, for example, field inhomogeneities and scanner drift has not been fully explained [47].

In order to resolve these issues, additional studies are necessary. One approach would focus on one region, likely the occipital cortex as the majority of previous work has been used this region. The inclusion of multiple task-paradigms (i.e., including both event-related and block-designs, differences in timing to explore the effects of jitter etc.) to may assist in understanding the impact of the BOLD paradigm and the resulting inter-subject variability. Additionally, repeated measures would provide exploration of the differences in protocols noted above.

## Conclusions

The previously observed relationship between GABA levels and BOLD activation in both the occipital cortex and other regions of the brain was not replicated in the current study. The absence of correlation emphasizes the need to interpret correlative imaging studies with caution. As discussed, the BOLD signal is a surrogate for neural activity, incorporating many hemodynamic and metabolic characteristics [1], and GABA measured with edited MRS is reflective of total GABA rather than specific GABA inhibitory neurotransmission [5]. Therefore, these two measures are both somewhat removed from the neuronal activity and signaling that underlie previous interpretations and hypotheses concerning GABA and BOLD [7,8,15]. Additional hemodynamic and physiological interactions, not incorporated into current models, may obscure the relationship between GABA concentration and BOLD activity. While caution must be exercised in interpreting this null result, it is likely that the intuitively pleasing result of “more GABA, less BOLD” oversimplifies the complex physiological relationships in play.
